# Induction and transmission of oncogene-induced senescence

**DOI:** 10.1007/s00018-020-03638-0

**Published:** 2020-09-16

**Authors:** Nattaphong Rattanavirotkul, Kristina Kirschner, Tamir Chandra

**Affiliations:** 1 Chakri Naruebodindra Medical Institute, Ramathibodi Medical School, Faculty of Medicine Ramathibodi Hospital, Mahidol University, 111, Bang Pla, Bang Phli, Samut Prakan, 10540 Thailand; 2 Institute of Cancer Sciences, University of Glasgow, Glasgow, G61 1BD UK; 3 MRC Human Genetics Unit, MRC Institute of Genetics and Molecular Medicine, The University of Edinburgh, Edinburgh, UK

**Keywords:** Oncogene-induced senescence, Secondary senescence, Notch signalling, Juxtacrine senescence

## Abstract

Senescence is a cellular stress response triggered by diverse stressors, including oncogene activation, where it serves as a bona-fide tumour suppressor mechanism. Senescence can be transmitted to neighbouring cells, known as paracrine secondary senescence. Secondary senescence was initially described as a paracrine mechanism, but recent evidence suggests a more complex scenario involving juxtacrine communication between cells. In addition, single-cell studies described differences between primary and secondary senescent end-points, which have thus far not been considered functionally distinct. Here we discuss emerging concepts in senescence transmission and heterogeneity in primary and secondary senescence on a cellular and organ level.

## Introduction

Cellular senescence is a stress response programme that irreversibly and stably arrests the cell cycle following a variety of intrinsic and extrinsic stressors such as shortening of the chromosomal termini, DNA damage, oxidative stress, oncogenic and mitogenic stimuli [1–3]. Cellular senescence was first described by Hayflick and Moorhead, who observed that cultured human fibroblasts undergo a finite number of cell divisions, a phenomenon later widely known as the ‘Hayflick limit’ and termed as replicative senescence [4]. The limited replicative capacity of cells in vitro spontaneously suggests a model for ageing at the cellular level. It is now clear that this proliferative arrest is mainly driven by telomere attrition [5]. Initially dismissed as an in vitro artefact that bore no relevance to biological mechanisms in vivo, the concept of cellular senescence has stood the test of the past five decades and evolved to become relevant beyond the ageing context, with critical physiological functions in preventing tumour formation, remodelling of tissues during embryonic development, and promoting wound healing and immunogenic clearance [6].

In the replicative senescence model of proliferating human cells, successive telomere erosion in each cell division ultimately uncaps the free double-stranded chromosome ends, causing them to be recognised as DNA double-strand breaks (DSBs) and activating the DNA damage response (DDR) pathway [7, 8]. The telomere dysfunction-initiated DDR signalling engages the ataxia telangiectasia-mutated (ATM) kinase that subsequently stabilises the tumour suppressor protein 53 (TP53), leading to an upregulation of its target the cyclin-dependent kinase (Cdk) inhibitor (CDKN1A). The TP53/CDKN1A pathway implements a cell cycle exit by inhibiting the activity of cyclin E-CDK2 and allowing the hypophosphorylated form of the retinoblastoma (RB) tumour suppressor to enable G1 arrest [9]. Alternatively, senescence can be established independently of the TP53/CDKN1A axis through the p16/RB tumour suppressor pathway. The cyclin dependent kinase inhibitor 2A (CDKN2A*)* locus encodes p16 and ARF, with the former repressing CDK4/6 while the latter cross-talking with the TP53/CDKN1A network.

Unlike quiescent or terminally differentiated cells, senescent cells do not respond to mitogenic cues and are not specialised cells resulting from a developmentally differentiated program. Senescent cells display distinguishing characteristics morphologically and biochemically. They are generally characterised by an enlarged and flattened shape and robustly express the lysosomal enzyme β-galactosidase, detectable by senescence-associated b-galactosidase (SA-b-gal) staining at sub-optimal pH [10]. Increased levels of p16 are also commonly used as a marker of senescence in vitro and in vivo. Another prominent feature is the formation of senescence-associated heterochromatic foci (SAHF), a rearrangement of heterochromatin into discrete nuclear subdomains visible by 4′,6-diamidino-2-phenylindole (DAPI) staining. One of the most striking changes associated with senescence is the secretion of a suite of pro-inflammatory cytokines and chemokines, extracellular matrix proteins, growth factors and metalloproteinases as part of a senescence-associated secretory phenotype (SASP) [11]. SASP components attract immune cells for clearance of senescent cells, ensuring tissue homeostasis. Another emerging fundamental feature of senescent cells is accumulation of lipofuscin, insoluble granules formed from damaged and cross-linked proteins [12]. Lipofuscin accumulation reflects macromolecular damage and altered metabolism, both of which have been reported as hallmarks of senescence, and has been exploited for identification of senescence in vitro and vivo [13, 14] as well as proposed as the first screening step in a multi-marker algorithmic approach for senescent cell detection [15]. However, to date, none of the senescence markers are absolutely reliable or specific for the in vivo identification of all senescence types and different markers must be used in combination to conclusively recognise senescent cells. Discovering novel senescence biomarkers that are of universally biological interest is, therefore, an ongoing quest in the field. Overall, the most recent consensus from the International Cell Senescence Association (ICSA) summarises the four interdependent hallmarks of senescence as: (1) cell cycle arrest; (2) SASP; (3) macromolecular damage; and (4) deregulated metabolism [15].

### Oncogene-induced senescence: primary senescence

Among a plethora of senescence stimuli, intrinsic changes from activated oncogenes invoke not only an irreversible state of proliferative arrest but also a biologically meaningful response that curbs malignant transformation. This mode of senescence is referred to as oncogene-induced senescence (OIS), also considered as primary senescence. The first observation of OIS was described when an oncogenic form of *Ras*, *Hras*^*V12*^, was ectopically expressed in primary cell culture of human lung fibroblasts IMR90. Senescent growth arrest is also stably established in vivo with a notable example being the ectopic expression of *Ras* in mammary epithelial cells [16]. In contrast to replicative senescence, OIS occurs in a telomere erosion-independent manner and can engage both the TP53/CDKN1A and p16/RB networks. Other studies have shown that loss of TP53 or its regulator p19 in mice provokes *Ras*-induced cancer cell invasion, while the reactivation of TP53 suppresses tumour growth whilst displaying common senescence markers [17–19]. OIS cells commonly have increased expression of p16 and can escape from senescence and re-enter the cell cycle in the event of low p16 levels [20]. The maintenance of OIS is dependent upon the accumulation of RB, which acts downstream of p16 through repression of E2F-target genes associated with DNA replication [21]. Despite much evidence on the well-established involvement of TP53 and p16 in OIS, in certain cell types and contexts TP53 and p16 are not required for the initiation and maintenance of OIS [22, 23]. Discordant results were also reported in human mammary epithelial cells, which undergo *Ras*-induced senescence without engaging p16 and TP53 [24]. This seems to reflect the variability between cell types in undergoing TP53- or p16-mediated senescence.

Recent studies have implicated the NOTCH signalling pathway as one of the regulators of OIS. NOTCH signalling is an evolutionarily conserved pathway and was first described in cell-fate decisions during wing development in *Drosophila* [25]. The canonical NOTCH pathway relies on a NOTCH receptor expressed on the signal-receiving cell upon direct contact with its ligand on the adjacent signal sending cell. This ligand-receptor interaction eventually leads to cleavage of the NOTCH intracellular domain (NICD), a constitutively active form of NOTCH, which enters the nucleus to bind to a transcriptional complex containing Mastermind-like 1 (MAML1) and other factors to activate target gene expression. Network enrichment analysis and flow cytometry have revealed upregulation of NOTCH1 in OIS cells accompanied by induction of transforming growth factor-beta (TGFβ) [21]. However, the increased NOTCH activity during OIS was only transient while cells transition into the early phase of primary OIS. The latter phase of OIS establishment saw the return of N1ICD and NOTCH-1 target levels to resting activity. Strikingly, similar to oncogenic activation, ectopic expression N1ICD was shown to drive cell-autonomous senescence, in what is known as Notch-induced senescence (NIS) or primary NIS, in human fibroblasts with a distinct secretory phenotype and chromatin structure from that of primary OIS triggered by *Ras* activation [26, 27]. For example, primary NIS cells show a reduction of basal levels of the proinflammatory cytokine IL-8 and activation of a canonical NOTCH ligand, JAG1, as well as TGFβ1, while lacking SAHF formation [26, 27]. Overall, these pioneering studies of NIS clearly demonstrated the contribution of NOTCH signalling as a transient component of primary senescence. Given the diverse roles of NOTCH signalling in development, cell differentiation and cancer, it is of fundamental interest to investigate the functional significance of NOTCH in senescence, which remains to be established.

The physiological role of primary OIS remains clear and unequivocal: serving as an early, cell-intrinsic tumour-suppressive mechanism. Multiple studies in lung and pancreas have shown that conditional activation of oncogenic KRAS^V12^ led to neoplastic transformation in pre-malignant conditions that were accompanied by senescence markers [28]. Another in vivo study in mouse lymphoid cells demonstrated that NRAS overexpression triggered OIS that prevents full lymphoma progression [29]. The tumour-suppressive role of OIS is also evident in the mouse prostate following depletion of the tumour suppressor PTEN, but not when both PTEN and TP53 were lost in advanced prostate cancer [30]. Crucially, in vitro and in vivo experiments in human melanocytic naevi (moles) present one of the most intriguing findings showing OIS as a barrier to tumourigenesis [31]. Up to 80% of these naevi harbour the *BRAF*^*V600E*^ mutation, the most frequent mutation in melanomas. *BRAF*^*V600E*^-expressing melanocytes persist in the growth-arrested state and display several OIS markers such as upregulation of p16 and positive SA-β-gal staining [32, 33]. Still, they retain the ability to transform into malignancy if BRAF-induced senescence is silenced or reversed. Naevi are therefore benign lesions, that arrest stably for decades before the course of melanogenesis takes place, representing one of the best available examples of OIS in vivo. It is therefore of great biological interest to understand the tumour suppressor state and its genomic dynamics before cells escape from senescence and progress into full-blown melanoma. Examples of in vivo OIS are numerous and have been extensively reviewed elsewhere [34, 35].

## Non-cell-autonomous aspects of OIS (secondary senescence)

### Paracrine transmission of senescence

Paradoxically, the past few years have witnessed contentious challenges to the prevailing function and mechanism of OIS, revealing more diverse and sometimes contradictory roles of OIS than previously believed. Far from being a simple and terminal state of replicative cessation, OIS cells communicate and interact with each other, consistent with the fact that OIS cells remain metabolically active. Pioneering studies showed that OIS cells are able to self-amplify expression of their SASP components such as Intereukin 6/8 (IL-6 and IL-8) alongside other inflammatory molecules in an autocrine manner, by reinforced activation of nuclear factor kappa-light-chain-enhancer of activated B cells (NF-κB) and CCAAT-enhancer-binding protein beta (C/EBPβ) [36, 37]. Subsequent work then described the ability of senescent cells to engage in cell-to-cell communication, modifying their microenvironment via paracrine signalling and establishing a mode of senescence named secondary senescence [38]. When co-culturing OIS and proliferating cells, OIS cells induced a senescence phenotype in their growing neighbouring cells through locally secreted SASP molecules [38]. Quantitative proteomics and small-molecule inhibitor screens have identified complex inflammasomes and secretomes containing transforming growth factor beta (TGFβ), vascular endothelial growth factor (VEGF) and chemokine CC motif ligand 2 (CCL2) as modulators of paracrine senescence. Interleukin 1 (IL-1) signalling was further shown to be a key upstream regulator of the proinflammatory signalling cascade and inhibition of the NLR family pyrin domain containing 3 (NLRP3) inflammasome, which regulates IL-1β, led to the suppression of the SASP phenotype [38]. Additional findings have also pointed to the interplay between DNA damage, oxidative stress and paracrine senescence mediated by the release of IL-1 and TGFβ, with DDR signalling being shown to induce senescence in bystander cells [39].

**Fig. 1:**
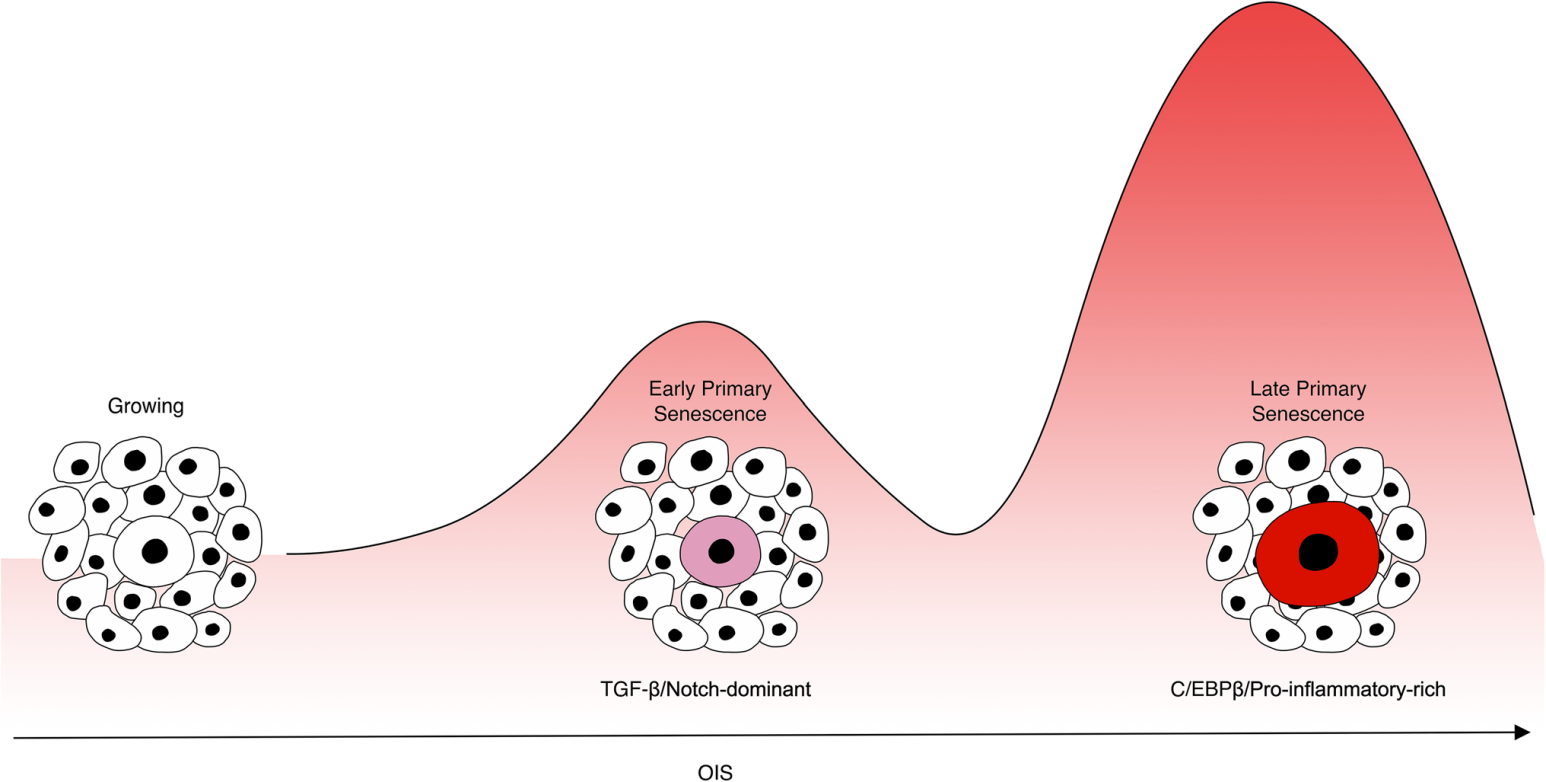
Schematic representation of the dynamic progression of primary oncogene-induced senescence. Primary OIS cells display two main phases, each with distinct a distinct SASP profile. The first or early stage of primary OIS is characterised by increased TGFβ signalling and NOTCH-dominant activity. The second or late stage of primary OIS is driven by C/EBPβ-rich and pro-inflammatory factors

The SASP secretome, as a mediator of paracrine activities of OIS, displays complex and dynamic features, part of which are regulated by NOTCH signalling [Fig. 1]. By comparing the transcriptomes and proteomes of NIS fibroblasts to those of the classic primary Ras-induced senescence (RIS), a time-course switch in the SASP-associated secretomes was revealed [26]. High levels of NOTCH1 drove a TGF-β-dominant secretome in the first wave of OIS secretion and at the same time inhibited a C/EBPβ-orchestrated SASP expression. The second wave was characterised by low levels of NOTCH1 and the reliance on C/EBPβ-dominant expression of proinflammatory cytokines. These two biochemically distinct and functionally opposing secretomes highlight the heterogeneity of SASP during the progression of OIS and the possible involvement of its spatio-temporal fluctuations in modulating paracrine transmission of senescence. What remain to be determined are the factors that control the switch of SASP dynamics and their physiological relevance.

As paracrine senescence is consistently observed in human and mouse models of OIS [38], the previously unappreciated non-cell-autonomous activities of OIS manifest a critical link between the expression of SASP factors and the induction of paracrine senescence in vivo, with far-reaching biological implications in determining the cellular outcome of normal cells in the context of tumourigenesis. By means of secreting SASP factors into the extracellular compartment, not only can OIS cells recruit immune cells to eliminate tumour cells [40, 41], but they can also promote tumourigenesis when physiological conditions become unfavourable. In skin, prostate and liver cancer models, the SASP phenotype is found to facilitate migration of cancer cells or favour an immunosuppressive microenvironment, which increases the risk of proliferation, angiogenesis and metastasis [42–44]. Taken together, it is becoming increasingly appreciated that OIS possesses a dual role in cancer pathogenesis, and this is largely dependent on the tissue context, the composition of the SASP and the duration of the senescence state. In this respect, there have been attempts to selectively remove non-advantageous senescent cells that may adversely transform their surrounding cells into malignant cells without affecting the functionally beneficial senescent cells. This has proved to be a success in a transgenic mouse system where p16-expressing senescent cells can be selectively eliminated [45]. Clearance of these p16-positive cells improved age-associated phenotypes, lifespan and tissue rejuvenation. A follow-up study then confirmed a significant reduction of spontaneous tumour formation after selective removal of senescent cells [46]. Paradoxically, a very recent report has demonstrated a slow process of senescence accumulation during a lifespan of p16-knock in mice whose p16high hepatic senescence cells appear to have important structural and functional roles [47]. Removal of liver cells with p16high led to liver and perivascular fibrosis, impairing the health of the animals. This suggests that the timing of senescence might be a key factor that determines the outcome of physiological and pathological conditions. Similarly, another observation also showed that C/EBPβ is a key player in the TGFβ cytostatic response by regulating expression of p15Ink4b, which contradicts the notion that OIS phase I involves TGFβ but not C/EBPβ [48]. It remains to be tested whether such contradictory results are physiologically relevant with the paradox observed in the elimination of senescent cells.

Hence, the concept of OIS as an in vivo antagonistic pleiotropy has emerged, featuring an anti-cancer action in early stages of oncogenesis and a switch to a deleterious, pro-cancer phase over time with advanced age. The key questions now needing answering are when and why exactly the twisted turn of senescence occurs. Pivotal to understanding the complexity of senescent effects is a comprehensive detail of how senescent cells interact with their microenvironment through short- and long-term secretion of SASP factors and accurate SASP factor composition.

More recently, the paracrine transmission of senescence has been found to play a role in cellular reprogramming, as shown when SASP components induced senescence and encouraged reprogramming by activation of octamer-binding transcription factor 4 (Oct4), SRY-box 2 (Sox2), Kruppel like factor 4 (Klf4) and MYC proto-oncogene, bHLH transcription factor (c-Myc) (OSKM or Yamanaka factors) in non-senescent cells [49, 50]. These studies collectively serve to improve our understanding of the complex network of the SASP, which contains many layers of regulation for signal amplification and exploits paracrine mechanisms to achieve secondary senescence in the cell neighbourhood both in vitro and in vivo. In addition, other modes of non-cell-autonomous activities of senescence have been described, including direct protein transfer through membrane protrusions connecting two cells called cytoplasmic bridges [51] and extracellular vesicles secreted from senescent cells [52, 53].

### Juxtacrine transmission of senescence

The molecular basis for controlling secondary senescence and the non-cell-autonomous properties of OIS are still under active investigation and revision, as the concept of secondary senescence is relatively novel and likely to be understood not as a static or permanent process, but as a highly dynamic and variable outcome depending on heterogeneous SASP secretomes. Coming into play is the contribution of NOTCH as a key mediator of contact-dependent secondary senescence, or juxtacrine senescence. Provided that the canonical action of NOTCH in mediating cell-to-cell signalling has critical implications in diseases such as cancer, the idea that senescence can spread by NOTCH1 signalling via direct cell-to-cell contact is an appealing notion that has only been pursued recently. As discussed earlier, the role of NOTCH signalling in senescence has been explored in NIS as a primary cell-autonomous process [21]. The same study also further revealed the non-cell-autonomous effects of NIS cells through a cell-contact dependent mechanism that propagates secondary senescence in their surrounding environment. Through ectopic expression of N1ICD, the primary senescent N1ICD-expressing cells were able to mediate lateral transmission of senescence to their neighbouring naïve cells in a JAG1-dependent manner. Inhibition of NOTCH signalling consequently compromised non-cell-autonomous secondary senescence. NOTCH-mediated juxtacrine senescence was further shown in a subsequent study to non-autonomously regulate chromatin structure in normal adjacent cells, repressing SAHF formation by suppressing expression of HMGA genes [23].

**Fig. 2:**
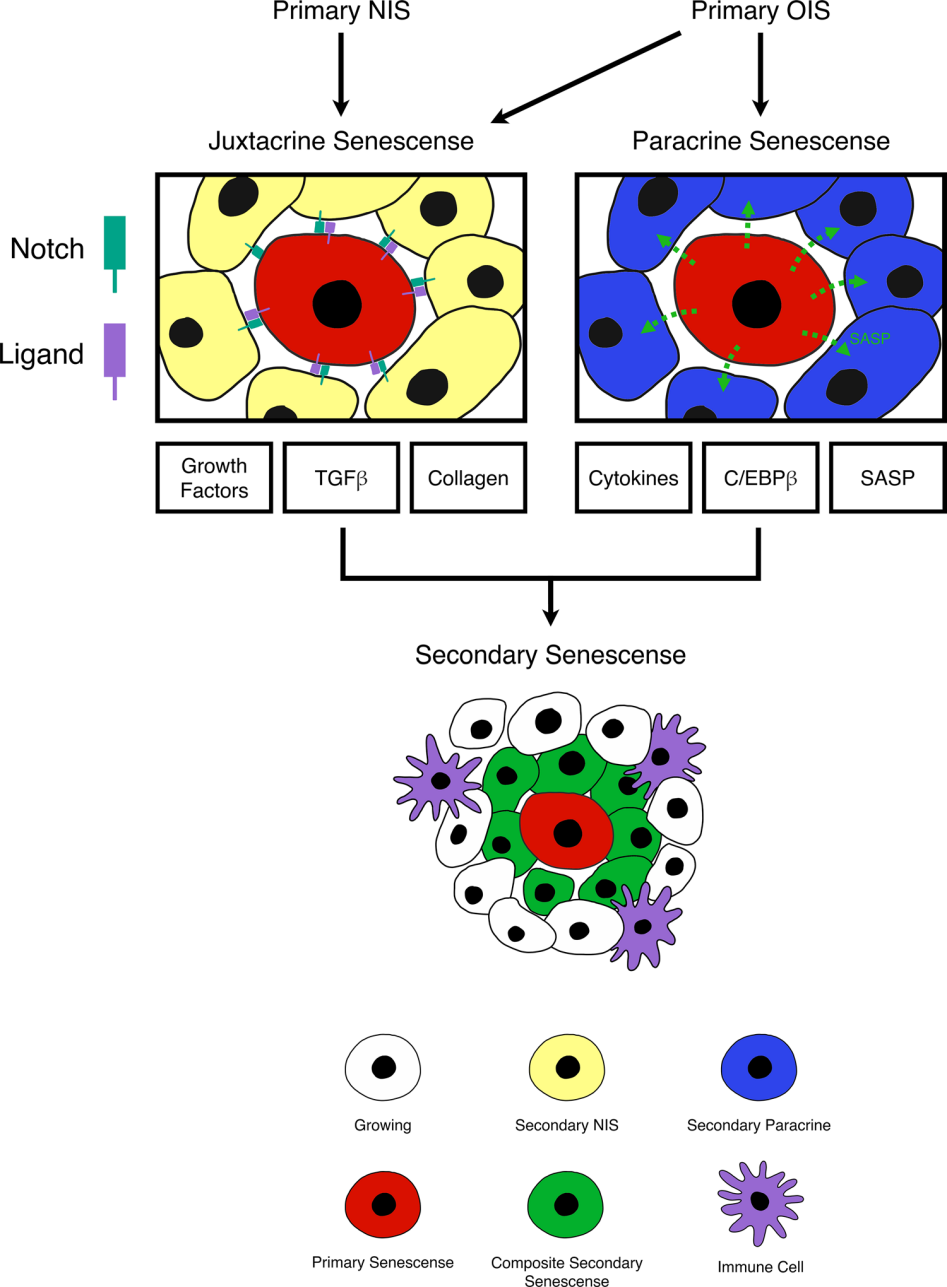
Schematic representation of how secondary senescence is established from primary senescence. There are two forms of primary senescence: oncogene-induced senescence (OIS) and Notch-induced senescence (NIS). These primary senescent cells can further trigger secondary senescence in their neighbouring cells in the process called secondary senescence, which can be mediated by cell-to-cell contact and/or paracrine transmission of senescence-associated secretory phenotype (SASP). Notch-dominant secondary senescence relies on expression of growth factors and TGFβ as well as the induction of fibrillar collagens. SASP-dominant secondary senescence, on the other hand, is driven by release of proinflammatory cytokines, expression of C/EBPβ and spread of SASP. Both Notch-driven and SASP-driven secretome profiles are found to contribute to a population of secondary senescent cells. Under normal conditions, the combined consequences of paracrine and juxtacrine signalling include the recruitment of immune cells and clearance of senescent cells. Here, primary and secondary senescent cells are viewed as functionally distinct endpoints

Despite the clear evidence of NOTCH signalling in facilitating juxtacrine secondary senescence, the nature of the secondary senescence population itself was not explored or characterised. The important question is whether NOTCH acts as a senescence inducer in neighbouring naïve cells that have not experienced oncogenic insults, in addition to SASP, particularly in the context of primary OIS cells that do not undergo ectopic activation of NOTCH. Recent work has presented a conceptual advance that primary and secondary senescence can be viewed as two distinct transcriptional termini and that NOTCH is an important effector of secondary senescence [54] [Fig. 2]. Single-cell RNA sequencing approaches were used to investigate the heterogeneity within OIS and identified two major transcriptional endpoints, one being a primary senescent population and the other a secondary senescent population. In contrast to the traditional view that paracrine senescence is the only mediator of secondary senescence in OIS, our downstream transcriptomic analyses revealed a pronounced enrichment of NIS signature in the secondary senescence population, suggesting NOTCH as a potent mediator. The requirement for NOTCH signalling for secondary senescence induction was confirmed in vitro through a co-culture system between NOTCH-incompetent cells and primary OIS cells. As opposed to the outcome of pure primary NIS, where the pro-inflammatory SASP components are suppressed and fibrillar collagens induced, the OIS-mediated secondary senescent cells showed both expression of SASP factors and induction of fibrillar collagens. Distinguishing the complex crosstalk between two modes of secondary senescence was only possible at single cell resolution. To further dissect the contribution of NOTCH to the activation of secondary senescence, secondary cells with impaired NOTCH signalling were co-cultured with primary OIS cells, and compared with secondary cells with intact NOTCH. Gene set enrichment analysis confirmed that the NOTCH-compromised cells upregulated the interferon-gamma response and pro-inflammatory factors relative to the normal secondary cells, suggesting that NOTCH signalling blunted the SASP response to a certain extent. Furthermore, this study provided in vivo data in secondary senescent-induced hepatocytes, which showed upregulation of NOTCH signalling compared to primary senescent hepatocytes. This concept was strengthened in an in vivo liver cancer model in which the recruitment of immune cells was compromised by the lateral transmission of NOTCH1-mediated senescence [26].

As research on secondary senescence is still in the nascent stage, the exact role and biological significance of NOTCH-mediated juxtacrine senescence and secondary senescence, in general, remain elusive. At least, it has become increasingly apparent that throughout the course of OIS, cells mature from pre-senescence to complete senescence, undergoing different stages where secondary senescence signals are dynamically transmitted to adjacent cells. Principally, secondary senescence encompasses both paracrine and juxtacrine modalities, but exact nature and timing of their operation and regulation is just beginning to emerge. In combination with the proposed model of the transient NOTCH/TGF-β-dominant secretome in the early phase of OIS and pro-inflammatory C/EBPβ-rich SASP in the late phase, the results from the single cell RNA-sequencing experiment of OIS cells suggest that juxtacrine senescence is predominantly achieved in the early NOTCH phase, while paracrine senescence takes on a more influential role when OIS is fully established [26, 27, 54]. The transition between the two phases is largely understudied, nevertheless. To understand whether and how secondary senescence is shaped by such a complex and dynamic programme, future investigation must include novel approaches that can dissect the relative contribution of each phase towards the induction of secondary senescence as well as determine their interactions with one another. Another tantalising area of study is to test whether the secondary paracrine wave of OIS also holds true in other senescence settings, from replicative senescence to DNA damage-induced senescence. The following question will then be asked: does the absence or presence of secondary senescence serve as a differentiating marker between OIS and other types of senescence, and does it have any physiological implications?

**Fig. 3:**
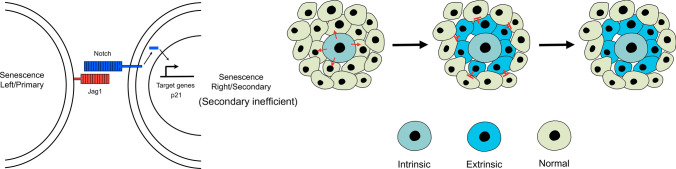
Schematic representation of the local containment of secondary senescence. Since the Notch ligand JAG1 is upregulated specifically in primary senescence cells, it could be possible that the secondary spread of senescent cells can be contained locally, as opposed to the senescence process expanding endlessly once established. Secondary senescent cells, which express low levels of the ligand JAG1, will not be able to induce further senescence responses. Such a local containment might allow unwanted senescent cells to be efficiently eliminated by the immune system

From a biological point of view, a conjectural interpretation of the composite transcriptional NOTCH and SASP signature in secondary senescence represents a fine control of cell proliferation activity and immune surveillance in the senescent milieu. Dysregulation of the initial stage of NOTCH-mediated senescence might promote tissue fibrosis through overexpression of collagen. Likewise, long-term persistence of inflammation or downregulation of NOTCH signalling in the late SASP-rich phase would in turn result in immunosuppression in the cellular neighbourhood and decrease the efficiency of immune-clearance of tumour-prone senescent cells, thereby favouring pre-neoplastic transformation in normal bystander cells [55]. In this context, alterations or disruption to the dynamic transition from the TGFβ-driven to the C/EBPβ-driven SASP secretomes might explain the dual and pleiotropic role of OIS in cancer formation. Therefore, under normal physiological conditions, the ultimate effects of juxtacrine senescence might lead to a scenario where the spread of senescence within a tissue is limited locally once secondary senescence is established [Fig. 3]. This local containment of senescence spread would guarantee tissue homeostasis and might be beneficial for immune cells to effectively eliminate detrimental senescent cells from that local environment. Verification of this functional model would be assisted by evidence in vitro and in vivo showing that NOTCH-enriched secondary senescent cells represent not a transient but a definitive transcriptional endpoint from which no further senescence is propagated and meaningful and effective immune-clearance can arise. Since primary senescence is widely recognised to suppress tumours, stimulate wound healing and play a role in the ageing process, it will be tantalising to investigate the function of secondary juxtacrine senescence in the contexts of tumour suppression, wound healing and ageing phenotypes in future studies.

### Transmission of senescence in organs

Senescent cell accumulation takes place in many tissues and organs, including the liver and the kidney, often in association with a functional decline and homeostatic capacity of those organs [56]. In hepatocellular senescence, the hepatocytes gradually lose regenerative potential and have increased susceptibility to chronic damage, cirrhosis, fibrosis and hepatocellular carcinoma [57, 58].

Compelling evidence suggests that hepatocytes undergoing primary senescence upon acute liver injury in the AhCre-Mdm2^fl/fl^ model can spread the senescence phenotype to nearby normal hepatocytes via SASP and the reliance on TGFβ signalling [52]. The TGFβ-dependent transmission of senescence was also reported in senescent cholangiocytes, which induced secondary senescence in normal surrounding hepatocytes while inhibition of TGFβ disrupted the transfer of senescence and restored liver function [59]. Combined with our recent in vivo liver data that revealed NIS signature in secondary senescent hepatocytes [54], it is possible that transmission of senescence in the liver is synergistically achieved by both paracrine induction and juxtacrine events.

The kidney is also known to be affected by constituents of SASP [60–62]. A variety of cell types in the kidney such as renal epithelial, endothelial, tubular and glomerular cells undergo age-associated changes over time with simultaneous upregulation of senescence markers [56, 63, 64]. Characteristics of aged kidneys include glomerulosclerosis, interstitial fibrosis and nephron atrophy, which contribute to an increased risk of chronic kidney injury and ultimately acute renal failure [65, 66]. In a mouse model of renal fibrosis, attenuation of p16 led to a decrease in interstitial fibrosis and nephron atrophy after ischemia–reperfusion injury, suggesting that inhibition of senescence could benefit renal function. This was further shown in transgenic mice where targeted ablation of p16-positive senescent cells attenuated glomerulosclerosis and helped to maintain healthy blood urea nitrogen levels [46]. Mounting evidence has shown that during chronic kidney disease SASP factors are secreted from endothelial cells and macrophages [60]. Consistently, a knockdown of plasminogen activator inhibitor-1 (PAI-1), a SASP factor, attenuated the formation of renal fibrosis upon kidney injury [67]. Interestingly, NOTCH signalling has also been implicated in senescent kidney cells. In mouse based study, the prolonged activation of tubular epithelial NOTCH1 diminished the regenerative potential of the kidney and was associated with the pro-senescent phenotype [68]. Yet not much is known about the establishment of secondary senescence by juxtacrine NOTCH signalling in the context of the senescent kidney. It will be useful and important to investigate the transcriptomes of senescent kidney cells and determine if primary and secondary senescence signatures can be distinguished and to what extent NOTCH and SASP contribute to secondary senescence.

## Concluding remarks

Oncogene-induced senescence unmistakably represents a tumour suppressor mechanism, but our understanding of its induction and establishment is not yet complete. The heterogeneity of the OIS programme is only beginning to be elucidated through the use of single-cell technologies. Among the key questions that need to be addressed are whether all cells respond similarly to the same oncogenic activation and whether the same oncogenic insult always results in a heterogenous OIS population. We have summarised current evidence for a functional heterogeneity in the senescence response in a recent review [69]. Studies of OIS cells and their transcriptomes in vitro and in vivo have unmasked a complex regulation of their dynamic and heterogeneous nature. Rather than being a stable entity following its triggers, OIS is endowed with both autonomous and non-cell autonomous activities, which allow OIS cells to interact with their neighbouring normal or transformed cells. One major effect of this interaction is the paracrine transmission of the senescent phenotype, largely mediated by secretion of a vast array of SASP factors, contributing to a secondary induction of senescence in the surrounding environment.

OIS, primary or secondary, is traditionally believed to have only one functional endpoint, but recent research has favoured the notion that primary and secondary senescent cells are in fact functionally distinct stages of OIS. Furthermore, transcriptomic analyses have challenged the prevailing view that paracrine induction of senescence is the only main mediator of secondary senescence in OIS, with juxtacrine NOTCH signalling emerging as a synergistic driver of secondary senescence. This means that OIS is a far more complex state than previously anticipated, with autocrine, paracrine and juxtacrine signalling pathways being at play. The precise mechanisms that govern the induction of senescence through paracrine or juxtacrine signalling, however, remain elusive. It is highly possible that the secondary induction of OIS is dictated by the dynamically spatiotemporal patterns of the senescence-associated secretomes as well as cell-to-cell interactions, which in turn shape the varied quality, quantity, output and changes of OIS functionality. To obtain a better insight into the molecular basis for the regulation of secondary senescence, novel technologies such as single-cell transcriptomics and genomics will prove valuable in identifying and characterising more subtle events taking place over the course of OIS, primary or secondary. Accordingly, investigating the heterogeneity of a senescent cell population is one approach to uncovering the actual contribution of each mode of senescence transmission, and will significantly highlight directions for the strategic development of new and specific drugs that target deleterious senescent cells.
